# Effectiveness of Smartphone-Based Physical Activity Interventions on Individuals' Health Outcomes: A Systematic Review

**DOI:** 10.1155/2021/6296896

**Published:** 2021-08-06

**Authors:** Mia A. Emberson, Anna Lalande, Danielle Wang, Daniel J. McDonough, Wenxi Liu, Zan Gao

**Affiliations:** ^1^College of Continuing and Professional Studies, The University of Minnesota, 208 Cooke Hall, 1900 University Ave. SE, Minneapolis, MN 55455, USA; ^2^School of Kinesiology, The University of Minnesota, Minnesota, USA

## Abstract

**Design:**

A systematic review. *Data Sources*. 114 studies were gathered using the following search descriptors: (“mobile phone” OR “smartphone” OR “cell phone” OR “mobile device” OR “mobile apps” OR “mHealth”) AND (“exercise” OR “physical activity” OR “physical fitness” OR “motor activity”) AND (“physiological outcomes” OR “weight outcomes” OR “psychological outcomes” OR “health” OR “health behavior”). Seven databases were used including databases such as Academic Search Premier and PubMed. PRISMA guidelines were followed in this review. *Eligibility Criteria for Selecting Studies*. The 20 articles included in this review met the following inclusion criteria: (1) randomized and controlled trials, (2) involving an outcome variable measured by accelerometer, and (3) intervention enforced by a smartphone application.

**Results:**

Overall, 56% of the studies reviewed in this paper resulted in successful interventions. Of the 19 articles that examined the first individual health outcome of physical and physiological outcomes, 11 interventions resulted in a positive effect on one of the following parameters: MVPA/step count, sedentary behavior, cardiorespiratory fitness, and blood pressure. Six interventions examined the effects on the second individual health outcome, weight-related outcomes. Five of these interventions observed significant positive effects from mobile application interventions on weight and waist circumference. Six articles evaluated the effectiveness of smartphone-based physical activity interventions on the third and final individual health outcome, psychological outcomes, with four resulting in significant positive outcomes in self-efficacy, life enjoyment/satisfaction, and intrinsic PA motivation.

**Conclusion:**

The findings in this review suggest that mobile application physical activity interventions, compared to unguided exercise activities, can effectively improve certain health outcomes for individuals such as physical/physiological and weight-related outcomes. It was found that research in the area of effectiveness of mobile application interventions on specific psychosocial health outcomes such as self-efficacy, life enjoyment, and intrinsic PA motivation is limited. Thus, the effect of mobile health applications remains unclear for psychosocial outcomes. Due to this limitation, more research is warranted to confirm the findings of this review.

## 1. Introduction

The health effects of physical activity (PA) are widely studied and have yet to produce wavering results—every individual can benefit from participating in physical activity. Data supporting the endless benefits of physical activity is widely available, yet approximately 80% of adults and adolescents in the United States do not meet the *Physical Activity Guidelines for Americans* (PAGA) [1]. This means that a very small fraction of Americans, likely less than 20%, are getting enough physical activity to reap the health benefits of physical movement. Thus, health professionals and researchers are committed to finding manageable ways for society to incorporate physical activity into daily routines. An area of research that is gaining popularity is integrating the use of mobile devices into physical activity regimens [2]. Following both a rise in popularity and the persistent nature of technology, smartphones and other mobile devices have been recognized as a method by which PA can be promoted. According to Sim [3], 81% of North Americans own a smartphone, making this device a very accessible outlet for promoting physical activity. Not only are smartphones widely owned but the always-on and accurate sensors built into the devices allow for reliable and easy-to-use tracking.

With the rise of technology and the growing obesity epidemic, evaluating the effectiveness of smartphone applications on specific individual health outcomes is just beginning to be a popular research area. For this reason, currently available literature is quite recent, and few meta-analyses exist to synthesize the literature that is available on these topics. Additionally, current research with mobile phone application interventions is often not specific nor comprehensive. Literature currently available is often general in the sense that interventions evaluate a general category of physical activity. For example, in a meta-analysis by Laranjo et al. [4], 25 of the 28 included studies found an increase in physical activity following an intervention carried out on an application on a mobile phone. Furthermore, recent studies have shown mHealth interventions to be effective at improving both mental and physical health outcomes in chronic medical conditions such as pediatric cancer [5]. The feasibility and acceptability of mHealth interventions have also been shown in patients with sickle cell disease [6]. Therefore, this review was conducted in order to create a comprehensive review of the available studies evaluating the effectiveness of mobile phone applications on specific individual health outcomes.

As previously stated, countless studies have shown regular participation in physical activity reaps numerous health benefits. Therefore, understanding methods by which to increase said physical activity behaviors is paramount to reduce the poor health outcomes resulting from a lack of physical activity. Few comprehensive reviews evaluating the effectiveness of mobile application PA programs on improving individuals' specific health outcomes are currently available. Thus, the purpose of the following review was to evaluate available literature assessing the effectiveness of smartphone-based PA programs on improving three categories of individuals' health outcomes—physical/physiological outcomes, weight-related outcomes, and psychosocial outcomes. The following review will outline and synthesize the currently available literature evaluating the effectiveness of smartphone-based physical activity interventions on individual health outcomes.

## 2. Methods

This review followed the Preferred Reporting Items for Systematic Reviews and Meta-Analyses (PRISMA) statement.

### 2.1. Information Sources

Databases such as Academic Search Premier, Communication and Mass Media Complete, Education Resources Information Center (ERIC), PubMed, Scopus, Web of Science, and Medline were used to search for articles used in the literature review.

### 2.2. Search Strategies

110 studies were gathered regarding smartphone-based physical activity interventions from 2013 to 2020 using the following search descriptors: (“mobile phone” OR “smartphone” OR “cell phone” OR “mobile device” OR “mobile apps” OR “mHealth”) AND (“exercise” OR “physical activity” OR “physical fitness” OR “motor activity”) AND (“physiological outcomes” OR “weight outcomes” OR “psychological outcomes” OR “health” OR “health behavior”). The literature search was conducted, and all appropriate studies were moved to a Google folder for further review of eligibility criteria.

### 2.3. Eligibility Criteria

Articles were included in this review if three main eligibility criteria were met: (1) randomized and controlled trials, (2) involving an outcome variable measured by accelerometer, and (3) enforced by a smartphone application.

### 2.4. Data Collection Process

Five reviewers (authors M.A.E., D.J.M., Z.G., A.L., and D. Y.) gathered the potentially relevant articles. Three reviewers (authors M.E., A.L., and D.Y.) then assessed the gathered articles and determined which articles met the inclusion criteria. Data was extracted from the relevant articles by two authors (A.L. and D.Y.) and was reviewed for accuracy by another author (M.E.). The following information was extracted from the relevant articles: (1) publication date and location; (2) specific groups involved; (3) study design (i.e., sample size, age range, study duration, treatment groups, and measurement instruments); and (4) key results and findings related to effectiveness of mobile intervention on physiological, weight-related, and/or psychological outcomes. Additionally, bibliographies of relevant articles were cross-referenced for additional studies by three reviewers (authors M.E., A.L., and D.Y.). These studies were then assessed by one reviewer (first author M.E.) to identify if the research was relevant to the literature review.

### 2.5. Data Items

The physical/physiological outcome variables included MVPA/step count, sedentary behavior, cardiorespiratory fitness, and blood pressure. The weight-related outcome variables included weight and waist circumference. The psychosocial outcome variables included self-efficacy, life enjoyment/satisfaction, and intrinsic PA motivation.

### 2.6. Risk of Bias in Individual Studies

Biases of each study were conducted by one reviewer (M.E.) through evaluation of nine quality assessment tools. As seen in Table 1, each category was given a “+” (positive) if it was clearly evident in the study or a “−” (negative) if the category was reported inaccurately or completely missing. Each study was given a score out of nine by totaling the “+” received from evaluation. If the study received a score of six or higher, the study could be evaluated as high quality with a low risk of bias. If the study received a score of five or lower, the study was evaluated as lower quality with a higher risk for bias.

### 2.7. Data Synthesis

The data collected from each article was evaluated, and each paper was put into a themed category of one of the three outcome variables. The results of each intervention were evaluated with relation to the outcome variable of the category they were placed in.

## 3. Results

### 3.1. Study Selection

**Figure 1:**
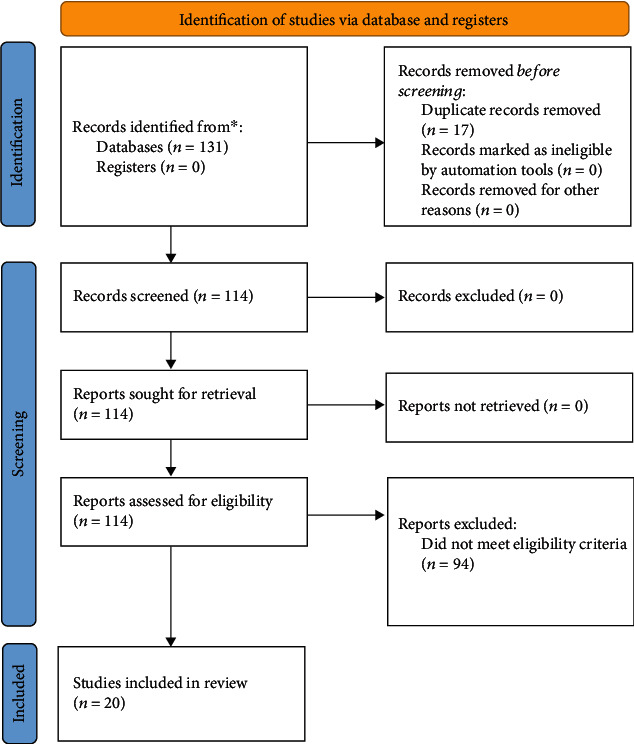
Flow diagram of studies through the review process.

An initial search of University of Minnesota Libraries and Google Scholar databases resulted in a total of 114 articles to be screened. After further evaluation for meeting inclusion criteria requirements, 20 articles were included in the following review. The study selection flow is shown in Figure 1. The included studies met the inclusion criteria of incorporating a smartphone/mobile application for the improvement of physical activity (PA) participation, psychological indicators, or medical conditions. Articles were excluded if they were (1) not randomized controlled trials, (2) did not use an accelerometer to measure outcomes variables, or (3) did not use a smartphone application.

Experiments were conducted from 2013 through 2019. Ten of the 20 studies were done in the United States in the states of California, Louisiana, Georgia, Pennsylvania, and Minnesota. The other ten studies were conducted in the following countries: New Zealand, Spain, the United Kingdom, Switzerland, Japan, the Netherlands, Belgium, and three in Australia. Sample sizes ranged from 30 participants to 833 participants and the ages of participants ranged from 12 years old to 70 years old. Most studies, however, involved middle-aged participants. Studies also involved specific groups such as individuals with type 2 diabetes, inactive pregnant women, overweight adults, young individuals, physically inactive females, African American women, breast cancer survivors, inactive college students, males in low-income schools, and patients with chronic obstructive pulmonary disease.

Studies (19) evaluating the first individual health outcome, physical and physiological outcomes, ranged from two weeks to twelve months evaluated outcome variables such as step count, sedentary behavior, cardiorespiratory fitness, duration of moderate to vigorous physical activity (MVPA), and blood pressure. Two of the 19 studies evaluated more than one of these variables of physical and physiological outcomes [2, 7]. The second research query investigated was weight-related outcomes (5). The studies for weight-related outcomes ranged in intervention time from 12 weeks to six months and were concerned with body weight and waist circumference. Three studies evaluated both weight and waist circumference outcomes [8–10]. The last grouping of studies focused on the effect of mHealth interventions on psychosocial outcomes including self-efficacy, life enjoyment/satisfaction, and intrinsic PA motivation (4). These interventions lasted between 12 weeks and 24 weeks. Two of the four studies evaluated more than one aspect of psychosocial outcomes [7, 11]. Some studies included in the review evaluated variables in more than one of the three large categories of individuals' health outcomes.

The mHealth intervention used varied among the studies. Two studies used the same Fitbit application for intervention [9, 12] and the other studies used one of the following applications: MoTHER, Zombies Run and Get Running, mPED, bActive, MyFitnessPal, Garmin, SmartLoss, Facebook, MyPlan 2.0, Moves, and ATLAS.

### 3.2. Quality and Risk of Bias Assessment

A risk of bias assessment was completed for each of the twenty studies included in the review. A visual display of the assessment is available in Table 1. The quality of the studies assessed ranged from a low score of four to a high score of eight out of the nine elements of evaluation. All studies but four received a bias assessment score higher than five points, giving a high-quality score to sixteen of the studies. The four studies that scored five or below were then consequently considered low-quality studies. All included studies had randomization procedures and included control groups. Most studies measured outcome variables before and after the study. Similarly, most studies retained more than 70% of participants. Unfortunately, less than half of the studies incorporated a six-month follow-up in the study design. Low scores on the risk of bias assessment can be credited to failures to conduct a power analysis as well as a lack of inclusion of validity measures.

### 3.3. Data Items

In this review, there were both physiological and psychological outcomes of interest. The characteristics of included studies are shown in Table 2. The outcomes were evaluated and divided into three categories: mHealth and physical/physiological outcomes, mHealth and weight-related outcomes, and mHealth and psychosocial outcomes. The physiological indicators for the mHealth and physical/physiological outcome category were MVPA/step count, sedentary behavior, cardiorespiratory fitness, and blood pressure. For the mHealth and weight-related outcome category, the indicators were weight and waist circumference. Lastly, the indicators for the mHealth and psychosocial outcome category were self-efficacy, life enjoyment/satisfaction, and intrinsic PA motivation. Conditions of interest in studies were type 2 diabetes, inactive pregnant women, overweight adults, young individuals, physically inactive females, African American women, breast cancer survivors, inactive college students, males in low-income schools, and patients with chronic obstructive pulmonary disease.

### 3.4. mHealth and Physical/Physiological Outcomes (MVPA/Step Count, Sedentary Behavior, Cardiorespiratory Fitness, and Blood Pressure)

Previous studies suggest that mobile phone applications can increase MVPA or step count in mobile application users. Nineteen of the 20 studies evaluated some aspect of the effect of mHealth on physical or physiological outcomes. Seventeen of the 20 studies included evaluated the effect of mobile app interventions on MVPA or step count. Of the 17 studies, a variety of outcomes were seen. Findings were mixed with one study showing a decrease in MVPA after intervention [13] and eight of the 17 studies showing no statistically significant effects of mHealth intervention on MVPA or step count [2, 7, 8, 11, 14–17]. The remaining eight studies showed a significant increase in MVPA or steps following the mHealth intervention [9, 12, 18–23], but one of these studies showed the findings dropped to neutral results at the six-month follow-up phase [18]. Thus, the findings on the effect of mHealth application on MVPA/step count are inconclusive.

Previous studies suggest that mHealth applications also decrease sedentary behavior among users. Three of the 20 studies evaluated sedentary behavior in relation to mHealth use, and two of the three studies indicated positive results [21, 24]. The third study that evaluated the effects of mobile application use on sedentary behavior showed no significant effects [16]. Ultimately, the findings on the effect of mHealth interventions on sedentary behavior are promising but inconclusive due to the mixed results.

Previous studies suggest that mHealth applications show promising effects on both increasing cardiorespiratory fitness and reducing blood pressure among intervention participants. The one study included in this review that evaluated cardiorespiratory fitness found that mHealth intervention resulted in no statistically significant findings [7]. The one study that evaluated blood pressure found that use of mHealth interventions resulted in a statistically significant decrease in systolic blood pressure among users following intervention [10]. Because only one study evaluated cardiorespiratory fitness and only one study evaluated blood pressure in this review, the effectiveness of mHealth on these variables cannot be determined.

### 3.5. mHealth and Weight-Related Outcomes (Weight and Waist Circumference)

Previous studies suggest that mHealth interventions can reduce body weight in mobile application users. Out of the 20 articles included in this review, five articles evaluated weight change as a result of intervention use. Three of the five studies showed that using mHealth interventions has a positive effect on weight-related outcomes, resulting in a decrease in body weight among participants [9, 10, 14]. Two of the five studies resulted in no statistically significant results, however both of these studies reporting in trending towards statistically significant weight loss [8, 25]. The results of mHealth intervention on body weight support the efforts of more research to uncover associations between mobile application intervention and weight loss.

Previous studies suggest that mHealth interventions can reduce waist circumference among participants. Three of the 20 studies included in the review evaluate the effect of mobile application interventions on waist circumference reduction. Of these three studies, two show statistically significant reduction in waist circumference as a result of participation in mobile application intervention [9, 10]. The other study shows positive trends towards reduction in waist circumference but does not present statistically significant results [8]. These results do not prove the effectiveness of mHealth intervention on waist circumference but continues to support further investigation of the effectiveness of mHealth on weight-related outcomes.

### 3.6. mHealth and Psychosocial Outcomes (Self-efficacy, Life Enjoyment/Satisfaction, and Intrinsic PA Motivation)

Previous studies indicate mHealth interventions can improve psychosocial outcomes such as self-efficacy. Two of the 20 included articles evaluate self-efficacy as an outcome variable, and the results are split. One study [11] shows that participation in mobile application interventions improves self-efficacy. Another study [7] shows mHealth interventions do not result in significant improvement of self-efficacy. Similarly, the results of the effects of mHealth interventions on life enjoyment are split. One study [7] shows no significant results following intervention while another [24] shows that mHealth interventions can improve life enjoyment.

Another area of psychosocial outcomes that previous studies indicate mHealth interventions can improve is intrinsic PA motivation. Two of the included articles evaluate the effect of mobile application interventions on intrinsic PA motivation [11, 20]. Both articles reviewing intrinsic PA motivation show that mHealth interventions are effective at improving intrinsic PA motivation.

Overall, the findings of the included articles that evaluate psychosocial outcomes show that mHealth interventions are not proven to improve self-efficacy nor life enjoyment. Intrinsic PA motivation, however, is shown to improve with participation in mHealth interventions. This idea is only supported by two studies, proving the need to investigate this further in future research.

## 4. Discussion

Physical activity has been deemed a productive method by which an individual can improve life from birth to old age, including but not limited to physical health, psychological health, and social wellbeing [26–30]. Thus, the following review was done with the main purpose of reviewing the current published literature evaluating the effects of physical activity on individuals' physical/physiological outcomes, weight-related outcomes, and finally on individuals' psychosocial outcomes.

### 4.1. mHealth and Physical/Physiological Outcomes (MVPA/Step Count, Sedentary Behavior, Cardiorespiratory Fitness, and Blood Pressure)

Among the studies that evaluated the effects of mobile interventions on step count/MVPA in the physical/physiological outcome category, eight resulted in positive results, eight resulted in no significant results, and one study resulted in negative results. Sixteen studies evaluating the effects of mobile application intervention on MVPA and step count in participants were split with half showing positive results and half resulting in no significant outcomes. At first glance, the overarching categories of the two groups of studies look very similar. Both the group of studies resulting in positive outcomes and the group of studies resulting in nonsignificant outcomes included studies from inside and outside the United States, both groups included studies with varying numbers of participants of all health conditions and ages, and both groups included studies of varying duration periods. After a closer look, however, there are two noticeable differences between the two groups of studies that found different results. First, 63% of the studies in the group that resulted in positive outcomes after intervention used a social comparison technique with participants [9, 18, 19, 22, 23]. This finding was supported by previous studies [4]. Among the eight studies that resulted in no significant effects after intervention, only 25% of the studies used a social comparison aspect in the study protocol [2, 14]. Second, among the eight studies finding success with the intervention, 75% of the studies did not include an aspect of physical activity education in the intervention [12, 19–23]. In contrast, 75% of the studies that did not have significant success with increased steps/PA after intervention did include education about the effects of exercise in the intervention protocol [2, 7, 8, 14–16]. The study that resulted in a decrease of MVPA among participants was carried out by Garcia-Ortiz et al. [13]. There are many aspects of this study to explain the negative results that were gathered. First of all, this study was the only included in the review that was carried out in Spain. Second, the participants in this study were all patients in primary care, another aspect unique to only this study included in the review. Third, the study included 833 participants—more than twice the participant size than any other study in the review. Fourth, the study duration lasted 12 months, which is twice as long as the next longest study included. Lastly, the study protocol included no aspect of feedback to participants during the duration of the study, something that nearly every other study included in the intervention protocol. The outcomes presented here with half of the interventions resulting in positive outcomes and half in nonsignificant outcomes were common in other meta-analysis [13, 31]. Romeo et al. [13] completed a similar meta-analysis and found that two of the five studies included in the review effectively improved physical activity in participants. In another similar review, Yerrakalva et al. [13] found that two of the four included articles in their meta-analysis produced positive outcomes for PA interventions.

Regarding the three studies that evaluated the effects of mobile interventions on sedentary behavior in the physical/physiological outcome category, two interventions resulted in positive results and one found no significant results after intervention. Both interventions that resulted in decreases in sedentary time used objective measures in the accelerometers to collect data on sedentary behavior [21, 24]. In contrast, the intervention that had no significant results used self-report measures to obtain sedentary behavior data [16]. This may prove to be the case because study participants are not fully aware of when they are engaging in sedentary behavior and when they are not. This would produce inaccurate data by self-report methods. Other studies have showed very similar results. A highly comparable meta-analysis by Stockwell et al. [32] included five studies, four of which had objective sedentary behavior measures and one of which had subjective measures. The four objectively collected data sets showed a significant decrease in sedentary behavior while the one study that used self-report measures showed no significant decrease in sedentary behavior.

One study included in this review evaluated the effect of mobile application intervention on cardiorespiratory fitness [7]. The mobile intervention in this study was not effective at improving cardiorespiratory fitness. These results were also found in a meta-analysis by Yerrakalva et al. [31] which evaluated three studies investigating the effect of mobile interventions on cardiorespiratory fitness. Yerrakalva et al. [31] presented that zero of the three included studies produced positive effects on cardiorespiratory fitness in participants. Similarly, only one study included in this review evaluated the effects of mobile interventions on blood pressure in the physical/physiological outcome category [10]. The intervention was successful and resulted in a reduced blood pressure in participants. A highly comparable meta-analysis by Gandhi et al. [33] included two studies that evaluated the effects of mobile health interventions on blood pressure—both of which were successful in decreasing blood pressure among intervention participants.

Looking at the physical/physiological outcome group as a whole, a few characteristics of successful interventions can be noted. Interventions that successfully increased the step count/MVPA of participants used a social comparison technique and did not educate study participants about the effects of exercise before the intervention period. Interventions that were successful in reducing participants' sedentary time used objective data collection measures such as always-on accelerometers. Similar to previous studies, the one study included in this review that evaluated cardiorespiratory fitness was not successful, indicating mobile interventions may not be effective in improving cardiorespiratory fitness. The included study evaluating blood pressure displayed a successful intervention, as did other studies evaluating mHealth on blood pressure present in previous meta-analyses. This may represent that any mHealth intervention can successfully reduce blood pressure.

### 4.2. mHealth and Weight-Related Outcomes (Weight and Waist Circumference)

Among the five studies that evaluated the effects of mobile interventions on weight in the weight-related outcome category, two studies found no significant results [8, 25] and three studies resulted in significant weight loss after the intervention [9, 10, 14]. Of these five studies, three also evaluated the effect of mobile application intervention on waist circumference. The results were the same as the weight outcomes, where Smith et al. [8] found no significant reduction after intervention but Bender et al. [9] and Martin et al. [10] did see significant results. This correlation makes sense because as the participants lose weight, their waist circumference will decrease as well. A reasonable explanation for why the successful studies were able to produce results is the length of the study. Two of the studies that resulted in a weight reduction after intervention had a study duration of six months [9, 14]. In contrast, both of the studies that did not have successful interventions had study durations shorter than six months [8, 25]. These results were also seen in a meta-analysis from 2019 evaluating the effects of mobile health on obese adults done by Park et al. [34]. This meta-analysis found that interventions lasting 6 months or longer had a much more significant impact on weight loss in participants. Another explanation for why some of the studies were successful while some were not is the age of the participants. The studies in which participants did not have success losing weight [8, 25] consisted of younger participants. Between the two studies, the oldest participant was 35 years old. For the studies that were successful with weight reduction, however, the average age of the participant was much older, with the youngest participant being 44 years old and going up all the way to participants who were 69 years old. A similar meta-analysis evaluating weight loss from mobile interventions [35] included six studies, four of which were successful in reducing the weight of participants. The four studies that were successful in reducing the weight of participants had an average participant age of about 45 years old, while the two unsuccessful studies involved participants of an age of 42 years old or less.

Looking at the weight and weight-related outcome group as a whole, a few characteristics of successful interventions can be noted. Interventions that successfully reduced the weight and waist circumference of participants were six months or longer in duration and had older participants, with an average age of around 45 years or older.

### 4.3. mHealth and Psychosocial Outcomes (Self-Efficacy, Life Enjoyment/Satisfaction, and Intrinsic PA Motivation)

Of the two studies that evaluated the effects of mobile interventions on self-efficacy in the psychosocial outcome category, one study found no significance [7] and one observed an increase in self-efficacy in participants after intervention [11]. A difference between these two interventions that may explain the contradictory outcomes is the use of short messages to participants in the intervention. Choi et al. [11] used various short messages to participants throughout each day of intervention with reminders that supported physical activity and other lessons. The intervention used by Direito et al. [7] used no such support messages during intervention. These messages could explain the difference in self-efficacy, where participants used the daily messages to encourage themselves to participate in physical activity. Without the messages, participants may have lost sight of the physical activity goals. Similar outcomes were seen in a meta-analysis by Aminuddin et al. [36]. This meta-analysis showed that SMS interventions were significantly more effective in improving self-efficacy scores than non-SMS interventions.

Two studies in this review evaluated the effects of mobile interventions on life enjoyment. One study showed no significant result after intervention [7], and one study found participants had an increased level of life enjoyment after completion of intervention [24]. One reason for the differing results could be due to the age of participants. Participants involved in the study done by Direito et al. [7] were much younger with an average age of about 15 years old, compared to the participants in the study by Kitagawa et al. [24] where participants were closer to 40 years old. Two studies included in this review evaluated the effect of mobile interventions on intrinsic physical activity motivation. Both studies resulted in improved intrinsic PA motivation as a result of the intervention [11, 20]. Both studies involved middle-aged participants who began the intervention period with less than perfect health, which may explain why the separate interventions were successful for both groups.

The results of the effectiveness of mHealth on psychosocial outcomes included in this review cannot be compared to other interventions because the availability of similar studies is extremely limited. At this time, the study of mobile intervention and its effects on life enjoyment and intrinsic PA motivation is in its infancy. Because of the mixed findings of the psychosocial interventions included in this review, future research is warranted to determine the effectiveness of mobile application interventions on psychosocial outcomes. Future research is also warranted in another newly emerging topic involving psychosocial effects of telemedicine during the COVID-19 pandemic. More recently, the availability and accessibility of mobile health care have risen dramatically due to social gathering restrictions. This accessibility, while the pandemic offered countless detrimental effects to individuals' health, may be a silver lining in which individuals will be able to access health professionals at anytime from anywhere [37, 38]. More research in future years will be necessary to understand the full effects of the pandemic on psychosocial wellbeing.

### 4.4. Strengths and Limitations

Overall, mobile application interventions prove to have promising effects on physical/physiological and weight-related outcomes, while the effect of mHealth on psychosocial health outcomes of participants remains unclear. Because all 20 articles included in the review were both randomized and controlled, the results can be considered valid. Additionally, many outcome variables were involved so the review is very extensive. However, a few limitations must be noted: (1) only studies published in English were included in this review, potentially excluding other research that has been done in this area; (2) a small fraction of all reviewed articles were included due to intense inclusion criteria; (3) the length of the intervention was not moderated in inclusion criteria and the relationship between intervention length and effectiveness was not examined exclusively; (4) the sample size of some included studies was small, limiting generalizability to larger populations. Also, important to note is that this review involved interventions provided through a smartphone. Based on the age of participants, the use of a smartphone may be viewed or implemented differently, potentially producing varying intervention results. This is important to keep in mind while creating a study protocol and during implementation of an intervention. Nonetheless, after reviewing the available literature on this topic, it can be concluded that mobile application interventions have the potential to improve physical/physiological and weight-related health outcomes. More research is warranted to prove effectiveness of mobile application interventions on psychosocial health outcomes.

### 4.5. Practical Implications and Conclusions

Based on the findings of this review, a few practical implications can be rendered for researchers and other health professionals. First, this meta-analysis closes important gaps in the currently available literature by relating the effectiveness of mobile health interventions to specific individual health outcomes. Second, new gaps in available literature were found as a result of this study. Future research is warranted to clarify the relationship between mobile health applications and psychosocial outcomes including self-efficacy, life enjoyment/satisfaction, and intrinsic PA motivation. Third, due to the successful nature of mobile application intervention's ability to increase steps/MVPA, decrease sedentary behavior, and reduce weight, utilizing these mobile interventions can be an important step in improving an individual's physical activity levels.

After reviewing the information included in this review, a successful proposed mobile application intervention used to increase steps/MVPA would include a social comparison aspect and would not brief participants with any educational aspect prior to participation. An intervention to successfully reduce sedentary behavior would use always-on accelerometers to collect movement data. A mobile application intervention that intends to successfully reduce weight or waist circumference of participants should have a duration of six months or longer. If these characteristics of studies are followed, mobile application interventions can be successful, thus increasing the physical/physiological as well as weight-related health outcomes of involved participants. There is, however, a lack of economic data to support the investment of mobile health applications and interventions. Badawy et al. [39] found that while technology-based interventions are gaining popularity, data showing the cost effectiveness while maintaining health outcomes is lacking. Another aspect of a successful intervention would have an aspect of patient preference, preferably early in the intervention. Badawy et al. [40] found that patient input is absolutely essential in ensuring both short- and long-term intervention adherence as well as enjoyment.

## 5. Conclusion

Due to the limited findings on some outcome measures, more research is warranted in the area of mobile application interventions on specific psychosocial health outcomes. It is proposed that in the future, researchers focus on the effects of mobile application intervention on physical health outcomes such as cardiorespiratory fitness, blood pressure, and psychosocial health outcomes such as self-efficacy, life enjoyment, and intrinsic PA motivation. From this review, it has been found that mobile application interventions can effectively improve certain health outcomes for individuals in some settings, but more research is needed.

## Figures and Tables

**Table 1:** Design quality analysis.

Articles	(1) Randomization	(2) Control	(3) Pre-post	(4) Retention	(5) Baseline	(6) Missing data	(7) Power analysis	(8) Validity measure	(9) Six-month follow-up	Score	Effectiveness
Bender et al. [9]	+	+	+	+	−	+	−	−	+	6	+
Choi et al. [11]	+	+	+	+	+	+	−	−	−	6	0
Direito et al. [7]	+	+	+	+	+	−	+	−	−	7	0
Fukuoka [18]	+	+	+	+	+	−	+	−	+	7	+
Garcia-Ortiz et al. [13]	+	+	+	+	−	+	−	−	+	6	−
Harries et al. [19]	+	+	+	+	−	+	−	−	−	5	+
Hartman et al. [14]	+	+	+	+	+	−	−	−	+	6	+
Hebden et al. [25]	+	+	+	+	+	+	−	−	−	6	−
Höchsmann et al. [20]	+	+	+	+	+	−	+	−	−	6	+
Kitagawa et al. [24]	+	+	+	+	+	+	−	+	−	7	+
Lynch et al. [21]	+	+	+	+	+	−	+	−	+	7	+
Martin et al. [10]	+	+	+	+	+	+	−	−	−	6	+
Melton et al. [15]	+	+	+	+	+	+	−	−	−	6	0
Patel et al. [22]	+	+	+	−	+	+	+	−	−	6	+
Patel et al. [23]	+	+	+	+	−	+	+	−	−	6	+
Pope et al. [2]	+	+	+	+	−	+	−	−	−	5	0
Poppe et al. [16]	+	+	+	+	+	+	−	+	+	8	0
Smith et al. [8]	+	+	+	+	+	+	+	−	+	8	0
Vorrink et al. [17]	+	+	+	−	+	−	+	+	+	7	0
Wang et al. [12]	+	+	+	+	+	+	−	+	−	7	+

Note: + refers to positive or present, − refers to negative or absent; retention = retaining more than 70% of the participants throughout the intervention; six-month follow-up = presence of a check in more than six months after the experiment.

**Table 2:** Characteristics of the included studies.

Study	Location	Study Description	Sample and Design	Measurement	Study Duration	Key findings
Bender et al. (2017)	San Francisco Bay Area, United States	Effect of PilAm Go4Health intervention with fitbit app on weight loss (secondary: steps)	45 total; 22 inintervention, 23 in control	Fitbit accelerometer	6 months	Statistically significant percent weight loss and fasting glucose change in type 2 diabetics
Choi et al. (2016)	San Francisco Bay Area, United States	Effect of MoTHER app (Mobile Technologies to Help Enhance Regular Physical Activity) on PA	30 total; 15 inintervention, 15 in control	Fitibt Ultra accelerometer	12 weeks	No significant difference between groups in steps or self reported PA , intervention saw higher self efficacy, significant evidence that intervention reduced lack of energy as barrier in inactive pregnant women
Direito et al. (2015)	Auckland, New Zealand	Effect of Zombies, Run and Get Running mobile app on cardiorespiratory fitness	51 total; 17 in immersive app, 16 in nonimmersive app, 18 in control group	Actigraph accelerometer	8 weeks	No significant findings between intervention and control group but time taken to complete fitness test decreased in both app groups compared to control
Fukuoka (2019)	San Francisco Bay Area, United States	Effect of mobile phone based PA education (mPED) on MVPA for 3 months as well as 9 month maintenance phase	205 total; 72 in regular, 60 in plus, 69 in control	Omron Active Style Pro HJA- 350IT accelerometer	9 months	3 month app and counseling intervention acheived significant increase in PA compared to control group for physically inactive females
Garcia- Ortiz et al. (2018)	Spain	Effect of app in addition to counseling on increasing physical activity (PA) and adherence to the Mediterranean diet	833 total; 415 in counseling+app, 418 in counseling	ActiGraph GT3X accelerometer	3 month intervention and 12 month follow-up	Overall found no differences between intervention group and counseling- only group in PA increase and adherence to the Mediterranean diet in the long term
Harries et al. (2013)	Bristol, UK	Effect of bActive app on step count	152 total	Accelerometer on smartphone app	6 weeks	Always-on, accelerometer-based smartphone apps can increase walking amongst males by around 64%
Hartman et al. (2016)	San Diego, California, United States	Effect of MyFitnessPal on MVPA and weight	54 total; 36 in intervention, 18 in usual care	ActiGraph GT3X Accelerometer,	6 months	Combining technology-based self- monitoring tools with phone counseling supported weight loss over 6 months in women at increased risk for breast cancer
Hebden et al. (2013)	Sydney, Australia	Effect of mHelath intervention with access to mobile app on body weight	51 total; 26 in intervention, 25 in control	ActiGraph GTIM accelerometer	12 weeks	Intervention and control group dropped weight, increased light intensity activity, and increased veggie intake
Hochsman n et al. (2019)	Basel, Switzerland	Effect of Mission: Schweinehund on intrinsic PA motivation (secondary: MVPA)	36 total; 18 in intervention, 18 in control	Accelerometer in Garmin Vivofit 2 activity wristband	24 weeks	Smartphone game significantly inmproved intrinsic PA motivation, leading to increased PA for inactive patients with type 2 diabetes
Kitagawa (2019)	Osaka, Japan	Effect of Smartphone application (UP) on sitting time (secondary: health related quality of life)	48 total; 16. in control, 16 in self feedback, 16 in tailored feedback	Jawbone UP24 accelerometer	2 weeks	All groups showed a significnat reduction in prolonged sitting time. For the tailored feedback group, the longest prolonged sitting time showed the most decrease following intervention.
Lynch et al. (2019)	Melbourne, Austrailia	Effect of Garmin app on MVPA levels	83 total; 43 intevention, 40 control	Accelerometer in Garmin Vivofit 2 wearable, activPAL	12 weeks	Wearbale technology in this study showed the ability to significnatly increase MVPA lveles in breast cancers survivors
Martin et al. (2015)	Baton Rouge, Louisiana, United States	Effect of SmartLoss app on weight, waist circumference (secondary: blood pressure)	40 total; 20 SmartLoss, 20 health education	Accelerometer A&D Engineering, Inc., Wellness Connected WirelessTM Activity Monitor XL-20	12 weeks	Showed signficant results in which SmartLoss particpants had significantly greater weight loss and reduction in waist circumerfance compared to Health Education for overweight adults
Melton et al. (2016)	Georgia, United States	Effect of Jawbone UP platform on physical activity	50 total; 17 intervention, 33 comparison	GT3X+ActiGraph activity monitor	6-week trial with 8-week follow-up	The physical activity intervention did not result in a signficant increase in physical activity for the intervention group compated to the control group
Patel et al. (2016)	Pennsylvania, United States	Effect of "Moves" app on step count using social comparison	286 total	Built in phone accelerometer	13 week intervention, 13 week follow-up	Found that social comparison (to median, 50% percentile) with financial incentives resulted in significantly more steps than other groups
Patel et al. (2018)	Pennsylvania, United States	Effect of "Moves" app on step count using lottery style	209 total	Built in phone accelerometer	13 week intervention, 13 week follow-up	Found that the combined lottery, which included both a higher frequency, smaller reward as well as a lower frequency, higher reward, was the most effect in increasing physical activity in overweigh adults
Pope et al. (2019)	Minneapolis, Minnesota, United States	Effect of Smartwatch+Facebook on intervention interest, use/acceptability, adherence, and retention (secondary: PA levels and diet)	38 total; 19 intervention, 19 contorl	Accelerometer- Polar M400 smartwatch	12 weeks	There was no significant advantage of intervention versus comparison
Poppe et al. (2019)	Ghent, Belgium	Effect of MyPlan 2.0 on PA and sendentary behavior	54 (RCT1)	ActiGraph accelerometer (GT3X=+)	5 weekly sessions with one week in between sessions for a total of 9 weeks	Study shows no significant positive effect for the ability of the intervetnion to increase PA or decrease sedentary behavior in type 2 diabetics
Smith et al. (2014)	New South Wales, Australia	Effect of Active Teen Leaders Avoiding Screen-time (ATLAS) on reducting obesity (secondary: physical activity)	293 total; 139 intervention, 154 control	ActiGraph accelerometer (GT3X=+)	20 weeks	Intervention was not successful in producing signficiant effects compared to control group for body composition but was for muscular fitness, movement skills, and weight related behaviors
Vorrink et al. (2016)	Netherlands	Effect of smartphone application on maintaining PA in COPD patients post 12 week COPD intervention	121 total; 62 intervention, 59 control	Accelerometer in smartphone (HTC desire A8181)	12 months	mHealth intervention did not improve or maintain physical activity in patients with COPD
Wang et al. (2016)	San Diego, California, United States	Effect of Fitbit One with mobile app on daily step count	67 total	ActiGraph accelerometer (GT3X=+)	6 weeks	Study showed that increased level of engagement with Fitbit One app was associated with increased steps

## Data Availability

The data used to support the findings of this study are available from the corresponding author upon request.
